# Biomaterial-Assisted Stem Cell Therapy and Exosome Delivery in Myocardial Infarction: A Narrative Review

**DOI:** 10.3390/biomimetics11080583

**Published:** 2026-08-15

**Authors:** Amanda-Ioana Răduţă, Andreea-Ramona Treteanu, Octavian Andronic, Ștefan Busnatu, Roxana Nicoleta Silişte, Elena Bălășescu

**Affiliations:** 1 Faculty of General Medicine, “Carol Davila” University of Medicine and Pharmacy, 050474 Bucharest, Romania; amanda-ioana.raduta0721@stud.umfcd.ro (A.-I.R.); andreea-ramona.treteanu0720@stud.umfcd.ro (A.-R.T.); stefan.busnatu@umfcd.ro (Ș.B.); roxana.siliste@umfcd.ro (R.N.S.); elena.balasescu@umfcd.ro (E.B.); 2 Center of Innovation and e-Health, “Carol Davila” University of Medicine and Pharmacy, 030167 Bucharest, Romania; 3 Department of Cardiology, Emergency Hospital Bagdasar-Arseni, 050474 Bucharest, Romania; 4 Colţea Clinical Hospital, 030171 Bucharest, Romania

**Keywords:** myocardial infarction, biomimetic biomaterials, stem cell therapy, exosomes, cardiac regeneration

## Abstract

Myocardial infarction remains a leading cause of heart failure because current reperfusion therapies cannot prevent adverse ventricular remodeling or restore lost cardiomyocytes. Regenerative strategies based on stem cells and extracellular vesicles (EVs) have emerged as promising approaches; however, their clinical efficacy is limited by poor retention, rapid clearance, and the hostile post-infarction microenvironment. This narrative review critically examines the role of biomaterial-assisted delivery systems in enhancing stem cell and EV-based cardiac regeneration, with particular emphasis on the distinction between biomimetic and bioactive biomaterials, mechanisms of action, preclinical and clinical evidence, translational barriers, and emerging regenerative technologies. Current evidence demonstrates that injectable hydrogels, extracellular matrix-derived scaffolds, cardiac patches, conductive biomaterials, and multifunctional delivery platforms improve therapeutic retention, prolong paracrine signaling, and actively modulate inflammation, angiogenesis, fibrosis, and extracellular matrix remodeling, resulting in superior functional recovery compared with conventional delivery approaches in preclinical models. Nevertheless, robust clinical evidence remains limited because few biomaterial-assisted strategies have advanced beyond early-phase studies. Future progress will depend on integrating smart biomaterials with engineered extracellular vesicles, gene editing, and personalized regenerative approaches, together with standardized manufacturing, harmonized regulatory frameworks, and adequately powered clinical trials.

## 1. Introduction

Cardiovascular diseases remain the leading cause of mortality worldwide, with ischemic heart disease representing the largest contributor to this burden [1,2,3]. Despite substantial advances in cardiovascular care, the global incidence and prevalence of myocardial infarction have nearly doubled over the past three decades, resulting in persistently high mortality, heart failure, and long-term disability [4].

Current management of acute myocardial infarction primarily aims to restore coronary perfusion through thrombolysis or percutaneous coronary intervention (PCI). Although early reperfusion substantially improves short-term survival, it does not prevent irreversible cardiomyocyte loss or the complex cascade of ischemia–reperfusion injury. Oxidative stress, inflammation, microvascular dysfunction, and extracellular matrix disruption continue to drive myocardial damage despite successful revascularization, ultimately promoting adverse ventricular remodeling and progressive heart failure [5,6].

Indeed, adverse remodeling develops in approximately 40–50% of patients following myocardial infarction, even after timely reperfusion and initially preserved left ventricular function [7]. Progressive ventricular dilatation, fibrosis, and contractile dysfunction remain major determinants of long-term morbidity and mortality, highlighting the inability of current therapies to adequately modulate the biological processes governing cardiac repair.

Several pharmacological strategies targeting post-infarction inflammation have been investigated; however, most clinical trials evaluating inhibitors of inflammatory pathways or broad-spectrum anti-inflammatory agents have produced inconsistent or only modest clinical benefits. Although therapies such as canakinumab and colchicine have demonstrated encouraging results in selected patient populations, their efficacy remains variable and insufficient to consistently prevent adverse ventricular remodeling or improve long-term outcomes [8]. These findings emphasize that myocardial repair cannot be achieved by targeting a single pathological pathway, supporting the need for regenerative approaches capable of simultaneously modulating inflammation, fibrosis, angiogenesis, and tissue remodeling.

Stem cell- and extracellular vesicle-based therapies have therefore emerged as promising regenerative strategies capable of restoring the injured myocardium through predominantly paracrine mechanisms. However, their clinical efficacy remains limited by poor retention, rapid clearance, low engraftment, and the hostile post-infarction microenvironment [9,10]. Persistent inflammation, oxidative stress, extracellular matrix remodeling, and fibrosis create an unfavorable niche that substantially compromises the survival and therapeutic activity of transplanted cells and extracellular vesicles.

These limitations have stimulated the development of bioactive biomaterials designed not only to deliver regenerative therapeutics but also to actively modulate the infarct microenvironment. Injectable hydrogels, extracellular matrix-derived scaffolds, epicardial cardiac patches, conductive biomaterials, and engineered exosome delivery systems improve local therapeutic retention while simultaneously providing mechanical support, controlled cargo release, immunomodulation, and structural cues that resemble the native myocardial extracellular matrix [11,12].

This review provides a critical overview of biomaterial-assisted stem cell and extracellular vesicle therapies for myocardial infarction, with particular emphasis on the biomimetic and bioactive principles underlying current delivery platforms. We discuss how biomaterials recreate the post-infarction microenvironment, enhance therapeutic retention, and improve regenerative efficacy, while critically examining the available preclinical and clinical evidence, the major barriers to clinical translation, and the future perspectives of biomaterial-assisted cardiac regeneration.

## 2. Materials and Methods

This study was conducted as a structured narrative review aimed at critically synthesizing current evidence on biomaterial-assisted stem cell and exosome delivery for cardiac regeneration following myocardial infarction. Rather than performing a quantitative synthesis, the review integrates experimental, translational, and clinical evidence to examine the biological rationale for biomaterial-assisted regenerative therapies, summarize the principal biomaterial platforms and delivery strategies, evaluate preclinical and clinical findings, and discuss current translational challenges and future perspectives.

A structured literature search was performed in PubMed, Scopus, and Web of Science without applying restrictions on publication date. The search was conducted using the following search strategy: (“myocardial infarction” OR “acute myocardial infarction” OR STEMI OR “ischemic heart disease”) AND (“stem cell” OR “mesenchymal stem cell*”* OR “MSC” OR “cardiac progenitor cell” OR “induced pluripotent stem cell” OR “extracellular vesicle” OR “exosome” OR “cell-free therapy”) AND (“biomaterial” OR “hydrogel” OR “injectable hydrogel” OR “scaffold” OR “cardiac patch” OR “extracellular matrix” OR “decellularized extracellular matrix” OR “biomimetic” OR “bioactive” OR “conductive” OR “cardiac tissue engineering” OR “controlled drug delivery” OR “regenerative medicine” OR “myocardial tissue engineering”). The search strategy was adapted to the indexing system and syntax of each database.

Peer-reviewed articles published in English investigating stem cell therapy, extracellular vesicles, biomaterials, or biomaterial-assisted regenerative strategies for myocardial infarction were considered eligible. Original experimental studies, clinical trials, systematic reviews, meta-analyses, and landmark methodological papers were included when they provided significant biological, translational, or clinical insights. Editorials, conference abstracts without full-text publication, duplicate reports, and studies unrelated to myocardial regeneration were excluded.

Study selection was performed independently by three authors in two stages. First, titles and abstracts were screened to identify potentially eligible publications. Subsequently, full-text articles were assessed according to the predefined eligibility criteria. Any disagreements regarding study eligibility were resolved through discussion and consensus among the reviewers. To minimize the risk of overlooking relevant evidence, the reference lists of all eligible publications were manually screened to identify additional relevant studies. A total of 111 references were ultimately included in the final manuscript.

Whenever possible, original research articles were preferentially cited to support mechanistic and experimental findings, whereas review articles were mainly used to provide broader conceptual background and contextualize the available evidence.

During manuscript preparation, OpenAI ChatGPT (GPT-5.5) was used to generate graphical icons for the figures. The scientific content, figure design, organization, annotations and final validation were entirely performed by the authors.

## 3. Pathophysiology of Myocardial Infarction

**Figure 1:**
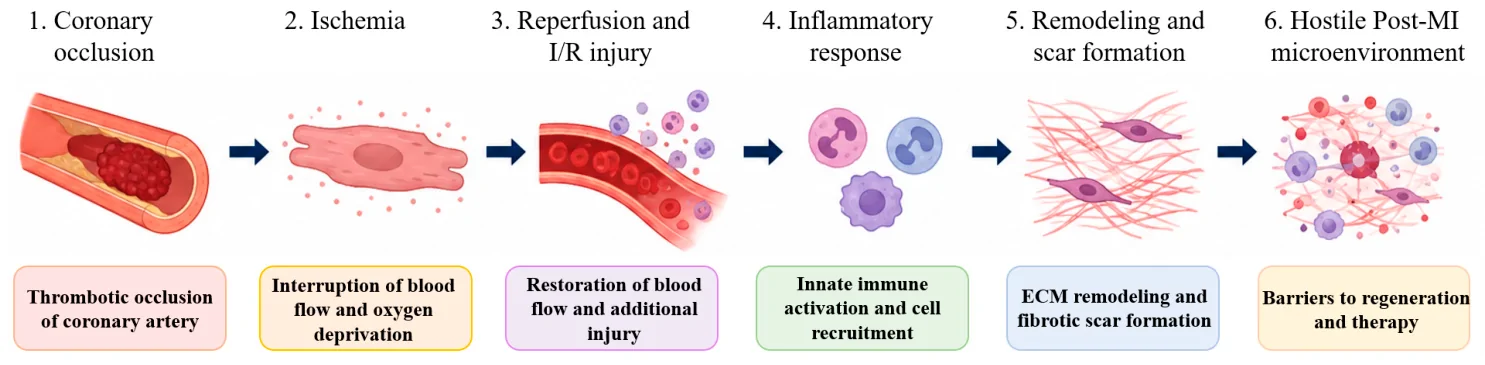
Major pathophysiological events following myocardial infarction. Abbreviations:* ECM, extracellular matrix; I/R, ischemia–reperfusion; MI, myocardial infarction*. *Graphical icons were generated using OpenAI ChatGPT (GPT-5.5), while the scientific content, organization, and annotations were developed by the authors.*

Acute myocardial infarction (MI) is most commonly initiated by thrombotic occlusion of a coronary artery following disruption of an unstable atherosclerotic plaque, resulting in abrupt interruption of myocardial blood flow [13,14,15] (Figure 1). Oxygen deprivation rapidly shifts cardiomyocyte metabolism from oxidative phosphorylation to anaerobic glycolysis, leading to ATP depletion, intracellular acidosis, calcium overload, mitochondrial dysfunction, and excessive generation of reactive oxygen species (ROS) [16,17]. If ischemia persists, irreversible cardiomyocyte death occurs. Although timely reperfusion remains the cornerstone of MI management, restoration of coronary blood flow does not fully prevent myocardial injury [18]. Instead, ischemia–reperfusion injury introduces an additional wave of oxidative stress, mitochondrial damage, microvascular dysfunction, and regulated cell death, further contributing to infarct expansion and functional deterioration [17,19].

Myocardial injury subsequently triggers a tightly coordinated but highly dynamic repair response. Necrotic cardiomyocytes release damage-associated molecular patterns (DAMPs), activating innate immune pathways and promoting the sequential recruitment of neutrophils, monocytes, and macrophages [16,20]. While this sterile inflammatory response is essential for debris clearance and initiation of tissue repair, its persistence or dysregulation amplifies extracellular matrix degradation, fibroblast activation, excessive collagen deposition, and adverse ventricular remodeling [16,21]. Progressive alterations in extracellular matrix composition, driven by matrix metalloproteinases, inflammatory mediators, and myofibroblast activation, ultimately replace functional myocardium with a mechanically stable but non-contractile fibrotic scar, predisposing patients to ventricular dysfunction and heart failure.

The hostile post-infarction microenvironment represents one of the principal barriers to successful cardiac regeneration. Persistent inflammation, oxidative stress, impaired vascularization, extracellular matrix disruption, and increased tissue stiffness markedly reduce the survival, retention, and functional integration of transplanted stem cells and extracellular vesicles [16,22]. Consequently, contemporary regenerative strategies increasingly focus not only on replacing damaged cardiomyocytes but also on modulating the infarct microenvironment. Biomaterial-assisted delivery systems have therefore emerged as promising platforms capable of improving therapeutic retention, recreating key structural and biochemical features of the native cardiac niche, and promoting a more favorable reparative response.

## 4. Stem Cell and Exosome-Based Mechanisms of Cardiac Repair

### 4.1. Evolution of Stem Cell Therapy

Stem cell therapy for myocardial infarction emerged from the recognition that conventional reperfusion and pharmacological strategies, although life-saving, do not replace the large number of cardiomyocytes lost after ischemic injury and therefore do not prevent progressive ventricular remodeling in many patients [23]. The earliest translational wave was driven by the idea that implanted cells might repopulate scarred myocardium and restore contractile tissue, an expectation that initially positioned skeletal myoblasts and subsequently bone marrow-derived cells as the first clinically accessible candidates. Menasché’s early clinical experience with autologous skeletal myoblast transplantation marked a symbolic starting point for human cardiac cell therapy and opened a period of rapid diversification toward other adult cell sources [24]. Soon after, the field shifted toward bone marrow-derived mononuclear cells, largely because they were relatively easy to harvest, autologous, and operationally compatible with post-infarction care pathways [25,26].

The randomized BOOST trial became one of the landmark studies of this phase, showing that intracoronary infusion of autologous bone marrow cells approximately 4.8 days after PCI increased global LVEF at 6 months by 6.7 percentage points versus 0.7 points in controls, without increasing restenosis, arrhythmias, or other adverse clinical events [27]. The improvement was most evident in myocardial segments adjacent to the infarct zone, suggesting that the therapy acted more by modifying the injured milieu than by rebuilding the infarct as new working myocardium.

However, the history of the field quickly became one of early enthusiasm followed by methodological and biological reassessment. Longer follow-up from BOOST showed that the initial gain in LVEF was not clearly sustained at 18 months or 5 years in the overall cohort, although patients with more transmural infarcts appeared to derive more durable benefit [28]. This pattern was reinforced by the placebo-controlled BOOST-2 trial, in which intracoronary nucleated bone marrow cells given about 8 days after PCI were safe but failed to improve LVEF significantly over placebo at 6 months; the estimated treatment effect for high-dose cells was only 1.0 percentage point, with a 95% confidence interval from −2.6 to 4.7 [29].

These clinical trajectories established a durable theme in the field: adult cell therapy after MI appeared feasible and generally safe, but its efficacy remained variable, context-dependent, and often limited to surrogate rather than unequivocal clinical endpoints.

At the same time, preclinical research broadened the therapeutic repertoire beyond naïve bone marrow mononuclear cells. Studies in small-animal infarction models reported benefit with cardiac-derived stem cells, adipose-derived stromal/stem cells, and mesenchymal stem cells, each improving ventricular performance or attenuating remodeling through distinct mixtures of trophic support, vascular effects, and limited lineage contribution [30,31].

In parallel, a second-generation strategy emerged: rather than delivering heterogeneous adult cells in their native state, investigators began trying to enhance reparative potency before implantation. A notable example was guided cardiopoiesis, in which bone marrow-derived human MSCs were preconditioned toward a cardiac progenitor-like phenotype, leading to superior structural and functional benefit compared with unguided cells in chronic infarction models [31].

More recently, biomaterial-supported delivery has been explored precisely because poor retention and engraftment were recognized early as major obstacles; hydrogel-encapsulated amniotic stromal MSCs, for example, showed improved persistence, neovascularization, and functional recovery in infarcted rat hearts [32].

### 4.2. Mechanisms of Stem Cell-Mediated Repair

Mechanistic thinking in cardiac cell therapy has changed profoundly over the last two decades. Early studies were built on the premise that transplanted adult stem cells might directly differentiate into cardiomyocytes and vascular cells, thereby replacing scar tissue with newly formed contractile myocardium. Although this possibility was never completely dismissed, subsequent work progressively showed that direct remuscularization is rare for most adult cell products and cannot by itself explain the functional improvements observed in experimental or clinical studies [26,30].

The strongest mechanistic consensus now supports paracrine signaling as the dominant mode of action. Adult stem cells release growth factors, cytokines, metalloproteinases, and extracellular vesicle-associated signals that can protect endangered cardiomyocytes, modulate inflammation, stimulate angiogenesis, and alter scar formation [26,33]. This conceptual shift also helps explain a long-standing clinical paradox: measurable benefit can occur despite poor long-term cell engraftment or limited myocardial homing after delivery.

**Figure 2:**
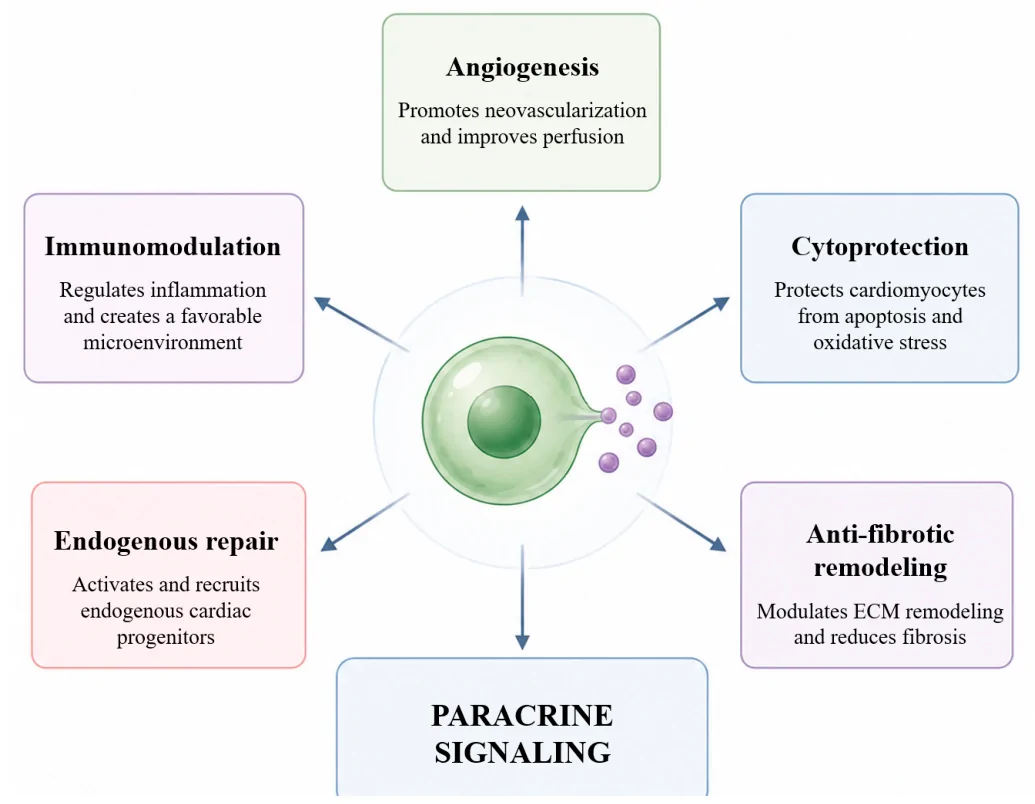
Mechanisms of stem cell-mediated cardiac repair. *Graphical icon was generated using OpenAI ChatGPT (GPT-5.5), while the scientific content, organization, and annotations were developed by the authors.*

Among paracrine effects (Figure 2), angiogenesis has been one of the most consistently demonstrated mechanisms. Mesenchymal and related stromal cells secrete VEGF, bFGF, IGF-1, HGF, and SDF-1-linked mediators that support endothelial growth, improve vascular density, and enhance perfusion of the peri-infarct myocardium [34]. In the infarcted heart, VEGF-modified MSCs increased myocardial SDF-1α expression, promoted mobilization and homing of bone marrow stem cells and cardiac stem cells, and improved ventricular function; blocking the SDF-1α/CXCR4 axis blunted these benefits, directly linking chemokine signaling to repair [34]. Likewise, cortical bone-derived stem cells expressed proangiogenic factors in vivo, notably bFGF and VEGF, and this was associated with increased neovascularization in the infarct border zone [25]. Adipose-derived stromal/stem cells also improved ventricular function while increasing peri-infarct arteriole density, despite showing little significant cardiomyocyte differentiation, again favoring a trophic-vascular mechanism [30].

A second major mechanism is cytoprotection, namely the preservation of endangered host myocardium rather than replacement of already lost tissue. Experimental work has shown that MSC-associated signaling can reduce apoptosis in cardiomyocytes exposed to hypoxic stress and can thereby attenuate adverse remodeling [25,33]. The study of Rap1/NF-κB signaling is particularly informative here: altering this pathway in bone marrow MSCs reduced stress-induced pro-inflammatory cytokine production, increased resistance of the transplanted cells themselves to hypoxia-induced apoptosis, and enhanced in vivo functional recovery while decreasing infarct size and inflammation [33]. These findings suggest that therapeutic efficacy depends not only on what cells secrete, but on how the inflammatory milieu shapes that secretome.

Immunomodulation and activation of endogenous repair programs appear to be closely linked to these cytoprotective effects. It has been proposed that transplanted cells can dampen local immune activation, repress ongoing myocardial apoptosis, and stimulate reparative host responses, including activation or recruitment of endogenous cardiac progenitors [26,35]. In guided cardiopoiesis models, transplanted cardiopoietic MSCs were associated not only with human cardiac protein expression in the recipient heart but also with upregulation of endogenous stem cells and increased host myocardial cell-cycle activity, supporting a combined exogenous-plus-endogenous repair model [31].

Collectively, current evidence supports a multicomponent mechanism of action in which paracrine signaling represents the dominant therapeutic pathway, while angiogenesis, immunomodulation, cytoprotection, anti-fibrotic remodeling, and activation of endogenous repair processes contribute synergistically to functional recovery after myocardial infarction. This mechanistic framework provides the biological rationale for biomaterial-assisted delivery strategies, which seek to maximize stem cell retention, survival, and secretory activity within the infarcted myocardium.

### 4.3. Biological Rationale for Exosome Therapy

Extracellular vesicles, particularly exosomes, have emerged as the principal mediators of stem cell-derived paracrine signaling and are increasingly regarded as a cell-free alternative capable of reproducing many of the regenerative effects of stem cell therapy. Their therapeutic potential derives from the transfer of proteins, mRNAs, microRNAs, lipids, and other bioactive molecules that modulate the behavior of recipient cells without the biological and logistical limitations associated with whole-cell transplantation [36,37].

This concept was supported by landmark studies demonstrating that exosomes can reproduce many of the beneficial effects of their parent cells while offering practical advantages such as lower immunogenicity, easier storage, and the potential for off-the-shelf administration [38,39]. Barile et al. showed that conditioned medium from human cardiac progenitor cells lost its anti-apoptotic and pro-angiogenic activity after extracellular vesicle depletion, whereas isolated vesicles fully reproduced these effects and significantly improved left ventricular function following myocardial infarction [37]. These findings established extracellular vesicles as active mediators of cardiac repair rather than passive by-products of cell secretion.

A complementary rationale derives from the biology of the injured myocardium itself. Following myocardial infarction, endogenous extracellular vesicles are actively released and exchanged between cardiomyocytes, endothelial cells, fibroblasts, and infiltrating immune cells, indicating that vesicle-mediated communication is an intrinsic component of the cardiac injury response [40]. Moreover, cardiac injury also releases exosomal miRNAs that appear capable of regulating migratory cell behavior and remodeling programs, further supporting the idea that therapeutic exosomes are not foreign to post-infarction biology but rather an attempt to redirect an existing communication system toward repair instead of maladaptation [41].

An additional advantage of exosome therapy lies in the possibility of engineering vesicle function before administration. Both hypoxic conditioning and pharmacological pretreatment of mesenchymal stem cells substantially modify exosomal cargo, enriching protective microRNAs and enhancing their regenerative activity [38,42]. Consequently, exosomes are increasingly regarded not only as natural mediators of stem cell signaling but also as programmable biological carriers whose therapeutic potency can be tailored according to clinical needs.

Despite these advantages, successful clinical translation remains dependent on overcoming several intrinsic limitations of native exosomes. Although they avoid many concerns associated with cell transplantation, including poor engraftment and tumorigenic risk, systemically administered exosomes exhibit rapid clearance, preferential accumulation within the liver and spleen, and limited myocardial retention [43]. These limitations have driven the development of targeted delivery strategies based on cardiac-homing peptides, ischemic myocardium-targeting ligands, and biomaterial-assisted delivery platforms, which have become central to the next generation of exosome-based therapies.

### 4.4. Therapeutic Cargo and Mechanisms of Exosome Repair

**Figure 3:**
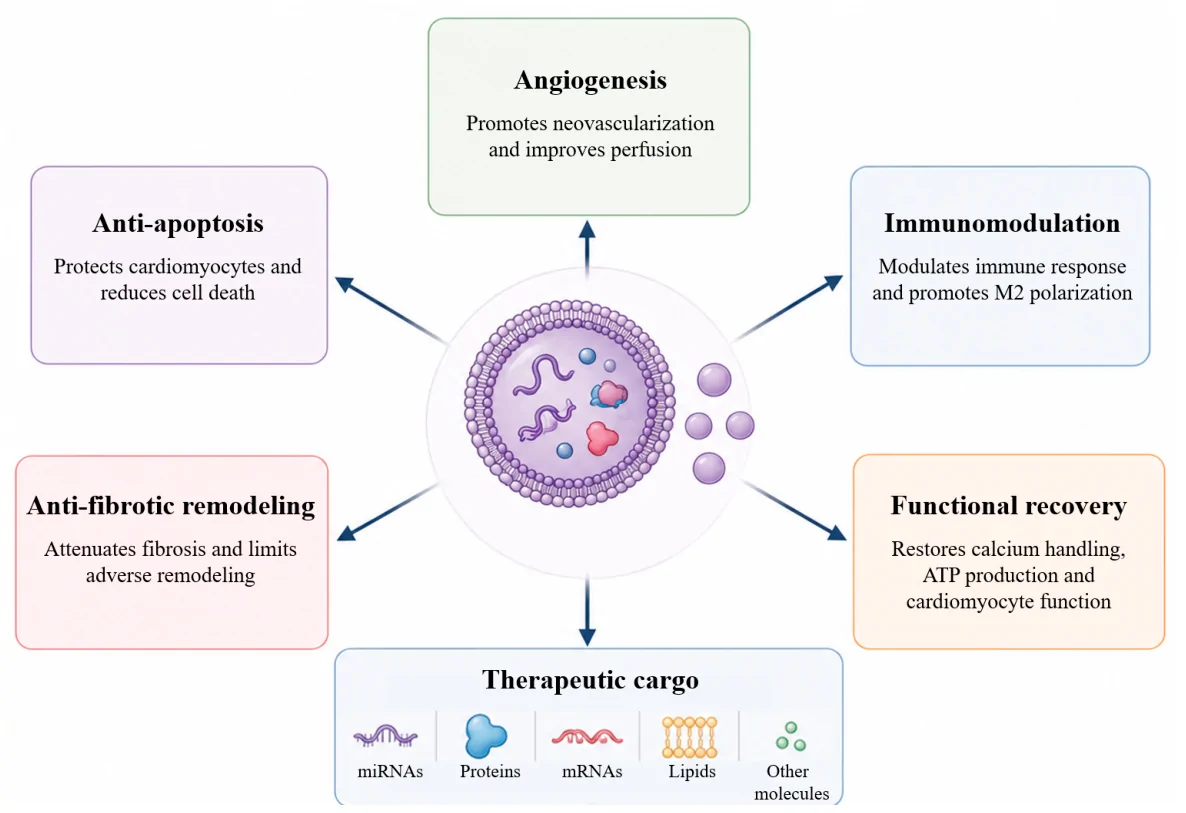
Therapeutic cargo and mechanisms of exosome-mediated cardiac repair. *Graphical icons were generated using OpenAI ChatGPT (GPT-5.5), while the scientific content, organization, and annotations were developed by the authors.*

The regenerative effects of exosomes are primarily determined by their molecular cargo rather than by the vesicles themselves. Although exosomes transport proteins, mRNAs, lipids, and other bioactive molecules, microRNAs represent the best-characterized mediators of cardiac repair (Figure 3). Rather than acting through a single signaling pathway, exosomal cargo simultaneously modulates apoptosis, inflammation, angiogenesis, calcium handling, fibrosis, and immune responses across multiple cardiac cell populations, including cardiomyocytes, endothelial cells, fibroblasts, and macrophages [43,44].

One major mechanism is anti-apoptotic cardioprotection. MSC exosomes enriched in miR-21a-5p transfer this miRNA to the myocardium and suppress several pro-apoptotic targets, including PDCD4, PTEN, Peli1, and FasL, establishing miR-21a-5p as a major mediator of MSC-derived cardioprotection [36]. A similar anti-apoptotic pattern was reported for hypoxia-conditioned MSC exosomes, in which enrichment of miR-125b-5p reduced infarct size and improved cardiomyocyte survival by suppressing p53 and BAK1 [38]. Additional studies extended this principle to miR-25-3p, which targeted FASL and PTEN and also reduced EZH2/H3K27me3 signaling to derepress eNOS and SOCS3, thereby coupling anti-apoptotic and anti-inflammatory effects [45].

Angiogenesis represents another major mechanism of exosome-mediated repair. Extracellular vesicles derived from human cardiac progenitor cells are enriched in miR-132, which promotes endothelial tube formation through downregulation of RasGAP-p120, while simultaneously reducing cardiomyocyte apoptosis via miR-210-mediated inhibition of ephrin A3 and PTP1B [37]. Likewise, M2 macrophage-derived exosomes improve cardiac function and reduce infarct size by delivering miR-132-3p to endothelial cells, where it suppresses THBS1 and enhances neovascularization [39]. More recently, inhaled stem cell-derived exosomes have identified endothelial cells as a principal therapeutic target, with single-cell analyses demonstrating downregulation of endothelial Cd36 and improved metabolic adaptation after myocardial infarction [46].

Another mechanism is immunomodulation, especially macrophage reprogramming. MSC exosomes reduced infarct size and inflammation after ischemia–reperfusion, and these benefits disappeared when macrophages were depleted, indicating that macrophages are not bystanders but required intermediates of repair [47]. In that setting, exosomal miR-182 shifted macrophages from an M1 to an M2 phenotype by targeting TLR4. A related study showed that nicorandil-pretreated MSC exosomes had stronger reparative effects because they increased miR-125a-5p and promoted M2 polarization through the TRAF6/IRF5 pathway [42]. Anti-inflammatory targeting also appears in engineered milk exosomes, where miR-146a delivery suppressed IRAK1/TRAF6/NF-κB signaling and reduced apoptosis and inflammatory cytokines after ischemia–reperfusion injury [48].

Beyond inflammation, exosomes regulate fibrosis, pyroptosis, and maladaptive ventricular remodeling. Exosomal miR-671 derived from adipose-derived MSCs attenuates fibrosis by targeting TGFBR2 and suppressing Smad2 phosphorylation [49], whereas miR-182-5p limits pyroptosis and infarct expansion through inhibition of GSDMD [50]. In a more comprehensive multicellular model, adipose stem cell-derived exosomal miR-196a-5p and miR-425-5p simultaneously enhanced angiogenesis, reduced mitochondrial dysfunction and reactive oxygen species generation, promoted M2 macrophage polarization, and, in the case of miR-196a-5p, inhibited myofibroblast activation and collagen synthesis, illustrating how a single exosome preparation can coordinate multiple reparative processes [44].

Finally, emerging evidence suggests that exosomes contribute not only to cell survival but also to functional restoration of cardiomyocytes. Exosomes derived from hiPSC-endothelial cells improved calcium transients, ATP production, and cardiomyocyte survival after oxygen-glucose deprivation while restoring SERCA2a and RyR2 activity through exosomal miR-100-5p, which targets PP-1β and increases phospholamban phosphorylation [51]. Conversely, exosomes isolated from cardiac stromal cells of patients with heart failure displayed impaired regenerative activity, whereas restoration of miR-21-5p rescued angiogenesis and cardiomyocyte survival via the PTEN/Akt pathway. These observations demonstrate that therapeutic efficacy depends not simply on exosome administration but on the precise composition of their molecular cargo [52].

Collectively, current evidence identifies exosomes as biologically active carriers of reparative information whose therapeutic efficacy is largely determined by donor-cell state, cargo composition, and delivery strategy. Consequently, the field is progressively moving beyond the administration of native vesicles toward the development of engineered exosomes with optimized molecular cargo, enhanced cardiac targeting, and improved regenerative potency. This evolution provides the biological foundation for the biomaterial-assisted delivery strategies discussed in the following sections.

## 5. Biomimetic and Bioactive Materials for Cardiac Regeneration

### 5.1. Biomimetic Versus Bioactive Biomaterials

In cardiac regeneration, the distinction between biomimetic and bioactive biomaterials is primarily conceptual, as most contemporary platforms integrate both properties. Biomimetic biomaterials are designed to reproduce the composition, architecture, and mechanical properties of the native cardiac extracellular matrix (ECM), thereby providing a permissive niche that supports cell survival, adhesion, differentiation, and tissue organization [53,54]. In contrast, bioactive biomaterials actively regulate the injured microenvironment through the controlled presentation or release of biological signals, including growth factors, cytokines, extracellular vesicles, or immunomodulatory cues, thereby directing tissue repair beyond passive structural support [55,56].

Biomimetic design is best exemplified by ECM-derived biomaterials, which preserve key biochemical and structural features of native myocardium. Decellularized cardiac ECM and cardiac-specific ECM scaffolds have been shown to improve cardiomyocyte maturation, promote three-dimensional tissue organization, and enhance cellular engraftment, emphasizing that effective biomimicry extends beyond collagen-based matrices to the recreation of tissue-specific extracellular complexity [57]. Nevertheless, native biomaterials alone rarely reproduce the full spectrum of biochemical and biomechanical signals required for functional myocardial regeneration.

These limitations have driven the development of bioactive biomaterials capable of actively instructing tissue repair. Modern hydrogel and cardiac patch platforms have been engineered to deliver angiogenic growth factors, regulate inflammatory responses, recruit endogenous reparative cells, and enhance the paracrine activity of transplanted stem cells and extracellular vesicles [55,58]. Rather than acting solely as passive scaffolds, these materials participate directly in angiogenesis, extracellular matrix remodeling, and immune regulation, thereby improving the regenerative microenvironment.

Consequently, biomimetic and bioactive properties should be regarded as complementary rather than mutually exclusive. The most advanced biomaterial platforms combine structural fidelity to the native cardiac ECM with instructive biological functions, enabling simultaneous restoration of tissue architecture and active modulation of the reparative response.

### 5.2. Recreating the Cardiac Microenvironment

Successful cardiac regeneration requires more than replacement of lost cardiomyocytes. It depends on reconstructing the complex myocardial microenvironment, which integrates extracellular matrix composition, mechanical properties, electrical conductivity, vascularization, and reciprocal interactions among cardiomyocytes, fibroblasts, endothelial cells, and immune cells. In the healthy heart, the extracellular matrix functions not only as a structural scaffold but also as a dynamic regulator of cellular behavior through biochemical and mechanotransductive signaling. Consequently, effective regenerative strategies aim to restore both the structural integrity and the biological instructions that govern myocardial homeostasis.

Among these components, the mechanical properties of the extracellular matrix play a central role. Following myocardial infarction, progressive fibrosis increases tissue stiffness and disrupts mechanosensitive signaling pathways that regulate cardiomyocyte proliferation and maturation. Experimental studies have demonstrated that biomaterials with physiologically relevant stiffness promote cardiomyocyte organization, angiogenesis, and functional recovery, whereas excessive matrix rigidity impairs regenerative responses [59,60]. Similarly, conductive biomaterials incorporating carbon-based nanomaterials or other electroactive components improve electrical coupling, sarcomere organization, and synchronous contraction by recreating the electrophysiological characteristics of native myocardium [61].

Reconstruction of the cardiac microenvironment also requires restoration of vascular and immunological homeostasis. Biomaterial-assisted delivery systems that promote controlled angiogenic signaling generate denser and more functional vascular networks, thereby improving oxygen delivery and long-term tissue viability [58]. At the same time, increasing evidence indicates that immune cells are active regulators of regeneration rather than passive inflammatory mediators. Biomaterials capable of modulating macrophage polarization and attenuating excessive inflammation create a more permissive niche for stem cell survival and tissue repair [62].

These findings indicate that the post-infarction microenvironment should be viewed as an integrated biological system rather than a collection of independent components. Effective cardiac regeneration therefore depends on simultaneously recreating the structural, mechanical, electrical, vascular, and immunological characteristics of the native myocardial niche, providing the biological foundation for biomaterial-assisted stem cell and exosome therapies.

### 5.3. Why Biomaterials Are Needed for Cardiac Regeneration?

The complex pathological microenvironment that develops after myocardial infarction largely explains why conventional regenerative therapies have achieved only modest clinical success. Although stem cells and extracellular vesicles possess considerable regenerative potential, their therapeutic efficacy is severely limited by poor retention, rapid clearance, inadequate engraftment, and reduced biological activity within the infarcted myocardium [63]. Consequently, only a small proportion of the administered therapeutic payload remains at the target site long enough to exert sustained reparative effects.

Biomaterials have emerged as an effective strategy to overcome these limitations by simultaneously addressing several barriers to cardiac regeneration. Beyond improving local retention of transplanted cells and extracellular vesicles, they provide temporary mechanical support to the infarcted myocardium, protect therapeutic cargo from premature degradation, and enable controlled, localized release of bioactive molecules [58,64]. These properties prolong paracrine signaling while reducing systemic biodistribution and off-target effects.

Contemporary biomaterials are increasingly recognized as active therapeutic components rather than passive delivery vehicles. By modulating inflammation, promoting angiogenesis, regulating extracellular matrix remodeling, and supporting electromechanical integration, they contribute directly to tissue repair while creating a permissive niche for endogenous and cell-based regenerative processes. Their therapeutic value therefore lies not only in enhancing delivery efficiency but also in actively reshaping the post-infarction microenvironment toward functional myocardial regeneration. The following sections examine the cellular and molecular mechanisms through which these regenerative strategies exert their reparative effects.

### 5.4. Major Biomaterial Platforms

A wide range of biomaterial platforms has been developed to overcome the structural and biological barriers that limit cardiac regeneration after myocardial infarction. Injectable hydrogels, extracellular matrix (ECM)-derived biomaterials, epicardial cardiac patches, conductive biomaterials, microcarriers, and nanoparticle-based systems represent the principal approaches currently investigated (Table 1). Although differing in composition and mode of administration, these platforms share the common objective of improving therapeutic retention while recreating key structural and biological features of the native myocardial microenvironment.

## 6. Emerging Regenerative Strategies

Despite considerable advances in biomaterial-assisted stem cell and exosome therapies, complete restoration of myocardial structure and function remains elusive. Consequently, recent research has shifted toward next-generation regenerative strategies that seek not only to enhance therapeutic delivery but also to precisely control cell fate, gene expression, paracrine signaling, and local tissue remodeling. These emerging approaches, including cellular reprogramming, gene editing, engineered cell-free therapeutics, and smart biomaterials, reflect a transition from conventional regenerative therapies toward integrated and programmable regenerative platforms.

### 6.1. Cellular Reprogramming and Gene Editing

Direct cellular reprogramming has emerged as a promising alternative to exogenous cell transplantation by converting resident cardiac fibroblasts into induced cardiomyocyte-like cells (iCMs). Because fibroblasts constitute the predominant cell population within the post-infarction scar, their in situ conversion offers the dual advantage of reducing fibrosis while simultaneously generating new contractile tissue. Early studies using non-integrating Sendai viral vectors substantially improved reprogramming efficiency, generating nearly 100-fold more contractile iCMs than conventional retroviral systems while reducing the time required for the appearance of beating cells from 30 to 10 days [77]. These improvements translated into reduced fibrosis and enhanced cardiac function in preclinical MI models. Subsequent approaches based on MGTH overexpression confirmed authentic fibroblast-to-cardiomyocyte conversion associated with improved ventricular function [78], whereas more recent PHF7-mediated strategies demonstrated that efficient cardiac reprogramming may be achieved through single epigenetic regulators, highlighting the growing importance of epigenetic modulation in cardiac regeneration [79]. In parallel, cardiomyocyte-derived extracellular vesicles have been shown to further enhance direct reprogramming efficiency, largely through miR-133-mediated signaling, suggesting that combining cellular reprogramming with extracellular vesicle therapy may further improve regenerative outcomes [80].

Gene editing has further expanded the regenerative toolbox by enabling precise modulation of both therapeutic cells and the injured myocardium. Current CRISPR-based strategies pursue three complementary objectives: activation of endogenous cardiogenic programs, optimization of stem cell function through targeted gene editing, and direct in vivo genome modulation using extracellular vesicle-mediated delivery systems [81,82,83]. An important example is the simultaneous CRISPRa-mediated activation of Gata4, Nkx2.5, and Tbx5, which successfully reprogrammed cardiac fibroblasts into induced cardiovascular progenitor cells (ciCPCs), with more than 80% of cells undergoing phenotypic conversion followed by differentiation into both cardiomyocytes and vascular cells. Following transplantation, these ciCPCs reduced scar formation and improved cardiac contractile function [81]. Mechanistically, CRISPRa promoted chromatin remodeling by opening previously inaccessible genomic loci and establishing self-sustaining transcriptional networks, demonstrating that CRISPRa can function not only as a gene-editing tool but also as an epigenetic platform for cellular reprogramming [81]. These findings have subsequently been supported by additional proof-of-concept studies demonstrating endogenous activation of cardiogenic transcription factors in both murine and human fibroblasts. Wang et al. showed that CRISPRa-SAM-mediated activation of GATA4, HAND2, MEF2C, and TBX5 confirmed the biological feasibility of endogenous cardiac reprogramming, although conversion efficiency remained low despite robust gene activation [84]. Similarly, Huang et al. demonstrated that endogenous activation of Gata4 could partially replace exogenous transcription factor delivery, but complete CRISPRa-mediated reprogramming remained inefficient and highly dependent on cell type, with substantially lower activation efficiencies in cardiac fibroblasts than in embryonic fibroblasts [85]. Importantly, neither study evaluated myocardial infarction models or functional cardiac repair, highlighting that current evidence primarily supports biological feasibility rather than therapeutic efficacy. Collectively, these studies provide convergent evidence supporting the biological feasibility of CRISPRa-SAM-mediated cardiac reprogramming. However, reprogramming efficiency remains variable and highly dependent on cell type and experimental conditions, while the available evidence is still limited to preclinical proof-of-concept studies. Additional independent validation and reproducibility across myocardial infarction models will be required before this strategy can be considered for clinical translation.

Gene editing has also been explored to enhance the therapeutic properties of stem cells. CRISPRa-mediated activation of PPARGC1A increased mitochondrial biogenesis, ATP production, and energetic reserve in human cardiomyocytes, representing the first demonstration that activation of a single endogenous metabolic regulator can improve post-infarction cardiac function through metabolic reprogramming [86]. Similarly, TLR4 editing in human mesenchymal stem cells achieved a 68% editing efficiency, significantly reduced NF-κB activation and the secretion of pro-inflammatory and pro-fibrotic cytokines without affecting proliferation or migration, ultimately enhancing myocardial repair after transplantation [82].

More recently, extracellular vesicles have emerged as efficient biological carriers for CRISPR components. Cardiotropic extracellular vesicles loaded with Cas9 ribonucleoprotein complexes targeting miR-34a successfully reduced cardiomyocyte apoptosis and attenuated post-infarction myocardial injury, illustrating the convergence of gene editing with targeted biological nanodelivery [83]. Beyond regenerative applications, CRISPR activation has also been successfully achieved directly in postnatal cardiomyocytes in vivo, where Schoger et al. demonstrated precise, dose-dependent activation of endogenous cardiac genes using a cardiomyocyte-specific dCas9-VPR platform. However, activation of Mef2d induced hypertrophic remodeling and fibrosis, emphasizing that therapeutic efficacy depends critically on appropriate target selection and transcriptional dosage [87]. Despite these encouraging advances, efficient, tissue-specific, and clinically scalable cardiac delivery remains the principal challenge for the translation of CRISPR-based regenerative therapies.

### 6.2. Engineered Cell-Free Therapeutics

Engineered exosomes have emerged to overcome two major limitations of native extracellular vesicle therapy: limited cardiac targeting and insufficient control over biological cargo. Current strategies converge on the same concept: engineering the exosomal surface, the therapeutic cargo, or both to enhance accumulation within the ischemic myocardium while amplifying anti-apoptotic, pro-angiogenic, and anti-fibrotic effects [88].

A landmark study demonstrated that mesenchymal stem cell-derived exosomes engineered with an ischemic myocardium-targeting peptide (IMTP) through Lamp2b-IMTP fusion preferentially accumulated within the infarcted myocardium, resulting in reduced inflammation, apoptosis, and fibrosis together with enhanced vasculogenesis and improved cardiac function [88].

Subsequent studies further refined this strategy by combining cardiac targeting with therapeutic cargo engineering. Bone marrow mesenchymal stem cell-derived exosomes modified with a cardiomyocyte-specific peptide and loaded with miR-302 exhibited enhanced cellular uptake, promoted cardiomyocyte proliferation, attenuated apoptosis and inflammation, and significantly improved cardiac function in myocardial ischemia–reperfusion models [89]. Similarly, biomimetic nanovesicles derived from umbilical cord mesenchymal stem cell exosomes, functionalized with a cardiac homing peptide and loaded with placental growth factor (PLGF), preferentially accumulated within the infarct zone, stimulated angiogenesis and cardiomyocyte proliferation, and markedly reduced post-infarction fibrosis [90].

Engineering the intravesicular cargo represents another rapidly evolving strategy. Exosomes overexpressing growth differentiation factor-15 (GDF-15) inhibited cardiomyocyte apoptosis, promoted autophagy, reduced infarct size, and enhanced angiogenesis through mechanisms associated with TERT upregulation and AMPK activation [91]. Collectively, these studies indicate that exosomes are no longer viewed as passive carriers of paracrine signals but as programmable biological nanoplatforms in which tissue tropism, molecular cargo, and regenerative activity can be independently optimized to maximize therapeutic efficacy.

Beyond engineering exosomal targeting and cargo, recent efforts have focused on overcoming the practical limitations of cell-free therapies, particularly poor myocardial retention, limited production yield, and long-term storage. Lee et al. engineered iron oxide nanoparticle-loaded MSC-derived nanovesicles that enabled magnetic guidance, significantly increasing myocardial retention while reducing apoptosis and fibrosis and improving functional recovery [92]. Supporting these findings, a meta-analysis of 37 randomized preclinical studies reported improvements of 10.85 percentage points in left ventricular ejection fraction and 7.19 percentage points in fractional shortening, although substantial heterogeneity and potential publication bias remain important limitations [93].

The field has also progressed beyond extracellular vesicles toward completely acellular regenerative constructs. Huang et al. developed an artificial cardiac patch composed of decellularized porcine myocardial extracellular matrix and synthetic cardiac stromal cells, which retained biological activity after prolonged cryopreservation while reducing scar formation, promoting angiomyogenesis, and improving cardiac function in both rodent and porcine models [94]. These findings illustrate the transition from engineered extracellular vesicles to off-the-shelf acellular regenerative platforms with improved scalability and translational potential.

### 6.3. Smart Biomaterials and Multifunctional Regenerative Platforms

Smart biomaterials are increasingly designed not only to deliver regenerative therapies but also to actively modulate the infarct microenvironment. By combining controlled release with electroconductive, antioxidant, or immunomodulatory properties, these multifunctional platforms enhance the efficacy of cell-free therapies beyond simple cargo delivery [95,96,97].

Recent studies have focused on integrating extracellular vesicles within biomaterial platforms to improve their therapeutic performance. An injectable conductive hydrogel loaded with umbilical cord mesenchymal stem cell-derived exosomes prolonged local exosome persistence while enhancing intercellular coupling, angiogenesis, and cardiac function after myocardial ischemia–reperfusion injury [96]. Similarly, decellularized pericardial scaffolds carrying cardiac adipose tissue-derived extracellular vesicles promoted vascularization while reducing inflammatory cell infiltration [97]. In another approach, a polyurethane scaffold incorporating decellularized extracellular matrix, gallic acid, and extracellular vesicles simultaneously provided mechanical support, antioxidant activity, and sustained extracellular vesicle release, resulting in reduced oxidative stress, attenuated fibrosis, and improved cardiac function up to 8 weeks after myocardial infarction [95].

These findings highlight the transition from passive biomaterial carriers toward multifunctional regenerative platforms capable of simultaneously delivering therapeutic cargo and actively modulating the biological and mechanical environment required for myocardial repair.

## 7. Preclinical and Clinical Translation

### 7.1. Preclinical Evidence

Preclinical studies consistently demonstrate that biomaterial-assisted stem cell and extracellular vesicle (EV) therapies outperform conventional cell or EV administration alone across both small- and large-animal models. In the largest meta-analysis addressing biomaterial-assisted stem cell therapy, Khan et al. reported improvements in left ventricular ejection fraction (LVEF) of 7.98 percentage points in small animals and 10.49 percentage points in large animals compared with stem cell therapy alone, with hydrogels showing the greatest overall benefit [98]. Similarly, a recent meta-analysis of injectable hydrogel-based therapies reported mean improvements in LVEF of 8.87 percentage points in rats and 16.45 percentage points in mice, with extracellular vesicle-loaded hydrogels producing the most pronounced functional recovery [99].

Beyond improvements in ventricular function, biomaterial-assisted therapies consistently increase vascular density, reduce infarct size and collagen deposition, preserve ventricular wall thickness, and attenuate adverse remodeling across a wide range of experimental models [98,100,101]. These benefits appear to arise primarily from the ability of biomaterials to prolong local retention and provide sustained release of regenerative cargo.

The direction of benefit is remarkably consistent across both rodent and large-animal models, although the strength of evidence differs considerably. Rodent studies dominate the current literature and frequently report substantial improvements in cardiac repair [46,102,103], whereas large-animal studies remain relatively few and are often limited by small sample sizes [104]. Nevertheless, available porcine studies have largely confirmed the ability of biomaterial-assisted therapies to improve ventricular remodeling, increase vascularization, and reduce fibrosis, supporting their translational potential [97].

Despite these encouraging findings, the current evidence remains highly heterogeneous. Variations in animal species, infarction models, biomaterial composition, therapeutic cargo, delivery routes, dosing strategies, and follow-up duration make direct comparisons difficult and preclude identification of a clearly superior regenerative platform. Taken together, current preclinical evidence provides robust proof-of-concept for biomaterial-assisted cardiac regeneration; however, standardized experimental protocols and adequately powered large-animal studies remain essential before these promising results can be reliably translated into clinical practice.

### 7.2. Clinical Translation of Biomaterial-Assisted Regenerative Concepts

Despite encouraging preclinical results, the clinical translation of biomaterial-assisted regenerative therapies remains limited. To date, most clinical studies have evaluated direct administration of stem cells, whereas relatively few have incorporated biomaterial-based retention platforms or controlled delivery systems. Consequently, current clinical evidence primarily reflects the efficacy of cell-based therapies rather than the full therapeutic potential of biomaterial-assisted regenerative strategies (Table 2).

The first clinical evaluation of combined intracoronary and intravenous umbilical cord-derived mesenchymal stem cell (UMSC01) therapy was reported by Hsiao et al. in a phase I first-in-human study involving six patients with acute myocardial infarction. Patients received UMSC01 through sequential intracoronary administration followed by intravenous infusion, an approach designed to combine local myocardial delivery with potential systemic immunomodulatory effects. Treatment was associated with sustained improvements in left ventricular ejection fraction, marked reductions in NT-proBNP levels, and favorable functional recovery over 12 months, without treatment-related serious adverse events [105]. However, the extremely small sample size, absence of a control group, and limited follow-up preclude definitive conclusions regarding long-term efficacy or ventricular remodeling.

Subsequently, Attar et al. conducted a randomized, single-blind, phase II study including 65 patients, comparing single-dose versus double-dose MSC administration, based on the hypothesis that repeated delivery could amplify paracrine therapeutic effects. The results supported this hypothesis: only the double-administration regimen produced a significant increase in LVEF, a clear anti-remodeling effect, and a reduction in infarct size, whereas single-dose administration failed to induce relevant functional changes [106].

In contrast to the functional signal observed with MSC administration, randomized clinical trials evaluating intracoronary bone marrow mononuclear cells (BM-MNCs) have generally reported modest or inconsistent efficacy despite an acceptable safety profile. The BAMI trial [107], a multicenter randomized study evaluating intracoronary BM-MNC therapy after STEMI, was terminated prematurely because of insufficient recruitment and therefore lacked adequate statistical power to determine whether BM-MNC therapy reduced all-cause mortality. Although fewer hospitalizations for heart failure were observed in the BM-MNC group, no definitive conclusions regarding clinical efficacy could be drawn. Separately, the REGENERATE-AMI trial with five-year follow-up [108] showed that early improvements in left ventricular function and myocardial salvage did not translate into sustained reductions in major adverse cardiovascular events or long-term clinical benefit. Although BAMI and REGENERATE-AMI differed in design and patient populations, both illustrate the continuing challenges associated with BM-MNC therapy and provide a rationale for investigating regenerative approaches with greater biological potency, including mesenchymal stem cells, cardiac-derived cells, extracellular vesicles, and biomaterial-assisted delivery platforms.

In contrast to stem cell therapy, clinical translation of biomaterial-assisted regenerative strategies remains at a much earlier stage. Despite extensive preclinical evidence supporting injectable hydrogels, epicardial patches, decellularized scaffolds, and other biomaterial-based delivery systems, no adequately powered randomized clinical trials have yet demonstrated their efficacy after myocardial infarction.

Similarly, extracellular vesicle therapy has not yet progressed beyond early translational development. Although large-animal studies have demonstrated the feasibility and short-term safety of biomaterial-assisted EV delivery, together with improvements in ventricular function, infarct size, fibrosis, and vascularization, completed randomized clinical trials evaluating EV-based therapies after myocardial infarction are currently lacking [46,97]. Consequently, the clinical promise of engineered extracellular vesicles and biomaterial-assisted delivery platforms remains supported primarily by robust experimental evidence rather than definitive human data.

## 8. Challenges and Future Directions

**Figure 4:**
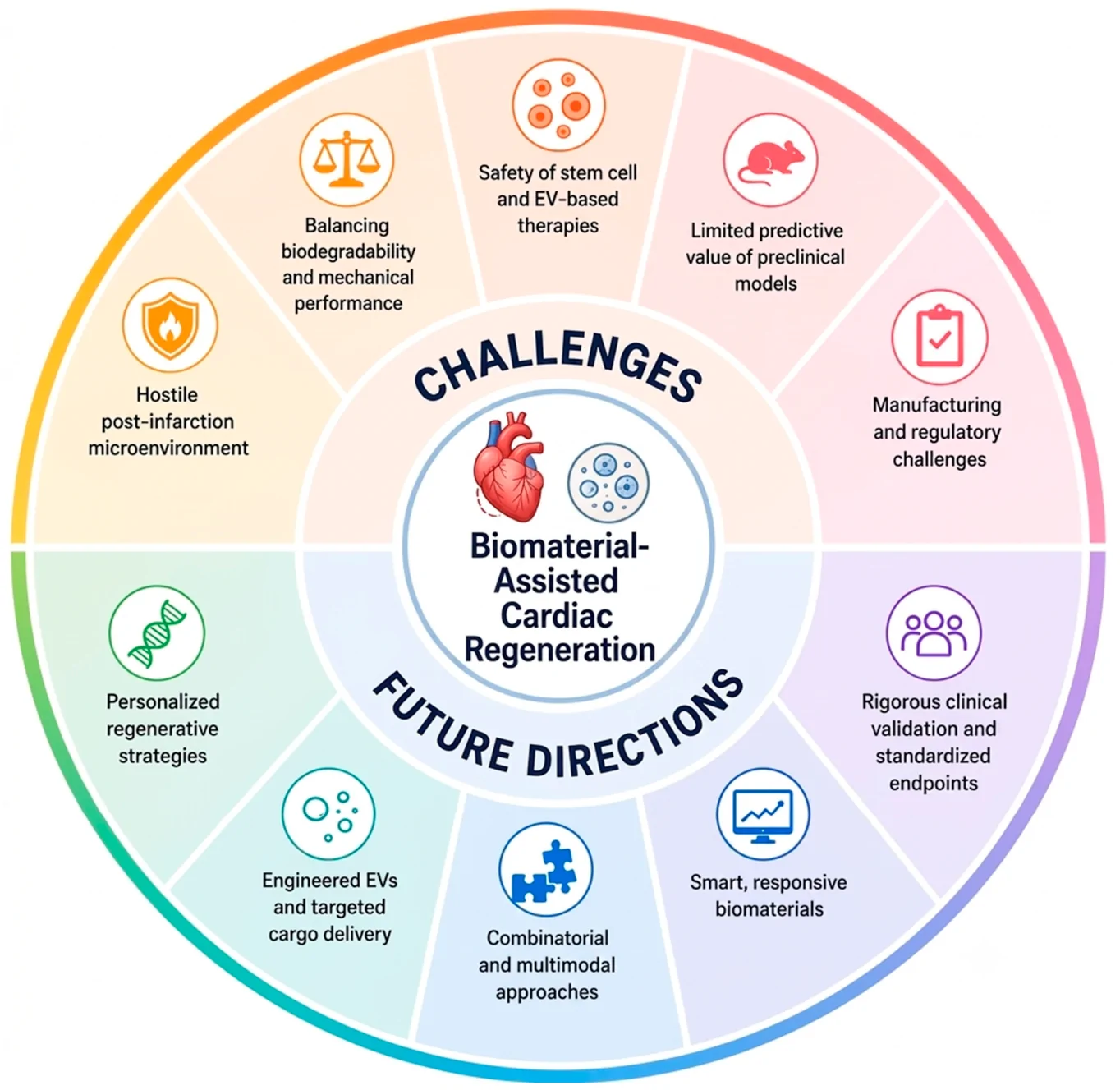
Major challenges and future directions in biomaterial-assisted cardiac regeneration. *This illustration was generated with the assistance of OpenAI ChatGPT (GPT-5.5) and subsequently reviewed and refined by the authors.*

A major biological challenge is the hostile post-infarction microenvironment, characterized by persistent inflammation, oxidative stress, extracellular matrix remodeling, and fibrosis, all of which compromise the survival and therapeutic activity of transplanted cells and extracellular vesicles (Figure 4). While biomaterials partially mitigate these limitations by improving local retention and providing structural support, current platforms still struggle to reproduce the mechanical, electrical, and biochemical complexity of native myocardium. Achieving the optimal balance between biodegradability, mechanical integrity, electrical conductivity, and long-term biocompatibility therefore remains a major engineering challenge. For pluripotent or cardiomyogenic products, additional concerns, including uncontrolled differentiation, tumorigenic potential, arrhythmogenicity, and ectopic tissue formation, continue to hinder the transition from transient paracrine effects toward durable myocardial remuscularization [109]. Furthermore, the predictive value of preclinical studies remains inherently limited, as most experimental models are performed in young, otherwise healthy animals with standardized infarction protocols, whereas patients with myocardial infarction typically present with advanced age, multiple comorbidities, chronic inflammation, and heterogeneous myocardial injury. These differences may substantially influence therapeutic efficacy and contribute to the persistent gap between encouraging preclinical findings and modest clinical outcomes.

Beyond these biological barriers, several manufacturing and regulatory challenges continue to hinder clinical implementation. Standardized large-scale production of biomaterials and extracellular vesicles under Good Manufacturing Practice (GMP) conditions remains challenging, while variability in extracellular vesicle isolation, characterization, potency assessment, and quality control continues to limit reproducibility across studies [109,110,111]. In addition, regulatory pathways for combination products integrating biomaterials with living cells, extracellular vesicles, or gene-editing technologies are still evolving, delaying clinical implementation.

Future research should prioritize the development of predictive preclinical models, standardized potency assays for cell- and extracellular vesicle-based products, and clinically relevant trial designs that enable meaningful comparison across studies. Integrating advances in biomaterials science with rigorous translational research will be essential to accelerate the clinical implementation of regenerative therapies for myocardial infarction.

## 9. Conclusions

Myocardial infarction remains a leading cause of heart failure because of the limited regenerative capacity of the adult myocardium. Although stem cells and extracellular vesicles have shown considerable therapeutic potential, consistent clinical efficacy has yet to be achieved despite encouraging preclinical findings.

The evidence reviewed in this manuscript indicates that biomaterial-assisted delivery represents a promising strategy to enhance the therapeutic performance of regenerative therapies and bridge the gap between experimental success and clinical application.

Overall, biomaterial-assisted regenerative therapies represent a rapidly evolving field with the potential to transform the treatment of myocardial infarction. Continued interdisciplinary collaboration between biomaterials science, regenerative medicine, and clinical cardiology will be critical to translate these advances into durable clinical benefit.

## Figures and Tables

**Table 1:** Overview of the major biomaterial platforms investigated for cardiac regeneration after myocardial infarction.

Biomaterial Platform	Biomimetic Features	Bioactive Features	Main Therapeutic Applications	Current Limitations	References
Injectable hydrogels	ECM-like hydrated matrix with tunable mechanical properties	Controlled release of cells, extracellular vesicles, and therapeutic molecules	Mechanical support, improved therapeutic retention, attenuation of adverse remodeling	Limited long-term mechanical stability; translational standardization remains challenging	[65,66,67,68]
ECM-derived biomaterials	Preserve native cardiac extracellular matrix composition and architecture	Retain endogenous matrix-associated biochemical cues that support tissue repair	Cell adhesion, angiogenesis, tissue remodeling, scar reduction	Batch variability, limited availability, and difficult standardization	[69,70]
Epicardial cardiac patches	Biomimetic fibrous or porous constructs providing structural myocardial support	Localized delivery of cells, extracellular vesicles, or bioactive molecules	Mechanical stabilization, enhanced therapeutic retention, myocardial repair	Surgical implantation and long-term integration remain limiting factors	[71,72]
Conductive biomaterials	Mimic the electrical properties of native myocardium	Promote electrical integration and enhance regenerative signaling	Improved electromechanical coupling, synchronized contraction	Balancing conductivity, biocompatibility, and mechanical performance remains challenging	[66,73]
Microcarriers	Three-dimensional biomimetic substrates for adherent cell attachment	Sustain cell viability and paracrine factor secretion	Enhanced cell engraftment, angiogenesis, sustained paracrine signaling	Manufacturing complexity and dependence on associated cell therapies	[64]
Nanoparticle-based systems	_	Targeted delivery of therapeutic cargo and modulation of endogenous repair pathways	Local delivery of bioactive cargo, immune modulation	Most evidence remains preclinical; long-term safety requires validation	[74,75,76]

**Table 2:** Clinical studies of regenerative therapies for myocardial infarction.

Study	Design	Therapeutic Approach	Main Findings	Key Limitations
Hsiao et al. [105]	Phase I, first-in-human, open-label, single-arm trial	Umbilical cord-derived mesenchymal stem cells (UMSC01) administered through sequential intracoronary and intravenous delivery	Improved LVEF, marked reduction in NT-proBNP, and favorable functional recovery over 12 months, with no treatment-related serious adverse events.	Very small sample size (*n* = 6), single-arm design, no control group, limited follow-up
Attar et al. [106]	Randomized, single-blind Phase II trial	Single versus repeated intracoronary MSC administration	Repeated MSC administration significantly improved LVEF, reduced infarct size, and attenuated ventricular remodeling; single-dose therapy showed no significant functional benefit	Moderate sample size (*n* = 65); longer-term efficacy remains to be established
BAMI trial [107]	Multicenter randomized clinical trial	Intracoronary autologous bone marrow mononuclear cells (BM-MNCs)	The study was underpowered because of insufficient recruitment and therefore no definitive conclusions regarding clinical efficacy could be drawn, although fewer hospitalizations for heart failure were observed in the BM-MNC group.	Limited efficacy despite acceptable safety profile
REGENERATE-AMI (5-year follow-up) [108]	Long-term follow-up of randomized trial	Intracoronary autologous BM-MNCs	Early improvements in LVEF and myocardial salvage observed at 1 year did not translate into improved long-term clinical outcomes, with no significant differences in MACE, recurrent myocardial infarction, repeat revascularization, or heart failure hospitalization after 5 years.	Demonstrated limited long-term regenerative efficacy of BM-MNC therapy

## Data Availability

No new data were created or analyzed in this study.
